# Whole Genome Sequences of the Wildtype AU-1 Rotavirus A Strain: The Prototype of the AU-1-like Genotype Constellation

**DOI:** 10.3390/v16101529

**Published:** 2024-09-27

**Authors:** Chantal Ama Agbemabiese, Francis Ekow Dennis, Belinda Larteley Lartey, Susan Afua Damanka, Toyoko Nakagomi, Osamu Nakagomi, George Enyimah Armah

**Affiliations:** 1 Department of Electron Microscopy and Histopathology, Noguchi Memorial Institute for Medical Research, College of Health Sciences, University of Ghana, Legon P.O. Box LG581, Ghanagarmah@noguchi.ug.edu.gh (G.E.A.); 2 Department of Hygiene and Molecular Epidemiology, Graduate School of Biomedical Sciences, Nagasaki University, 1-12-4 Sakamoto, Nagasaki 852-8523, Japan; tnakagom@gmail.com (T.N.); onakagom@nagasaki-u.ac.jp (O.N.)

**Keywords:** rotavirus A, AU-1-like genotype constellation, culture adaptation, Illumina MiSeq sequencing, reference genome

## Abstract

Most human rotaviruses belong to the Wa-like, DS-1-like, or AU-1-like genotype constellation. The AU-1-like constellation, albeit minor, captured attention because its prototype strain AU-1 originated from feline rotavirus, leading to the concept of interspecies transmission of rotavirus. The AU-1 genome sequence determined by various laboratories over the years has documented two conflicting VP7 sequences in the GenBank. As culture-adaptation may introduce changes in the viral genome, the original fecal (wild-type) and the seed stock of culture-adapted AU-1 genomes were sequenced using the Illumina’s MiSeq platform to determine the authentic AU-1 sequence and to identify what mutational changes were selected during cell-culture adaptation. The wild-type and culture-adapted AU-1 genomes were identical except for one VP4-P475L substitution. Their VP7 gene was 99.9% identical to the previously reported AU-1 VP7 under accession number AB792641 but only 92.5% to that under accession number D86271. Thus, the wild-type sequences determined in this study (accession numbers OR727616-OR727626) should be used as the reference. The VP4-P475L mutation was more likely incidental than inevitable during cell-culture adaptation. This was the first study in which the whole genomes of both wild-type and cultured RVA strains were simultaneously determined by deep sequencing.

## 1. Introduction

Rotavirus A (RVA), a member of the genus *Rotavirus*, family *Sedoreoviridae* [1], is a major cause of gastroenteritis in the young of humans and animals. Its genome consists of 11 segments of double-stranded RNA which encode six viral structural proteins (VP1–VP4, VP6, and VP7) and six non-structural proteins (NSP1–NSP5/6) [2]. Each genome segment has sequence diversity, and numbers are assigned to classify them into genotypes based on pre-defined nucleotide sequence identity cutoff values [3]. Thus, a unified classification system was adopted for the constellation of the VP7-VP4-VP6-VP1-VP2-VP3-NSP1-NSP2-NSP3-NSP4-NSP5/6 genes to be described as Gx-P[x]-Ix-Rx-Cx-Mx-Ax-Nx-Tx-Ex-Hx, where x denotes a genotype number [3]. 

Most human RVAs are largely classified into two major genotype constellations called Wa-like (G1/3/4/9-P[8]-I1-R1-C1-M1-A1-N1-T1-E1-H1) and DS-1-like (G2-P[4]-I2-R2-C2-M2-A2-N2-T2-E2-H2), and one minor constellation called AU-1-like (G3-P[9]-I3-R3-C3-M3-A3-N3-T3-E3-H3) [3]. The AU-1-like strains captured attention because the prototype strain, AU-1, was shown to have originated from rotaviruses circulating in cats [4,5]. This discovery led to the concept of interspecies transmission of rotaviruses as a mechanism driving the evolution of the rotavirus genome [6].

The full genome sequences of a vast majority of classical laboratory strains, including the prototypes that represent the three genotype constellations of human RVAs, were determined from the cell-culture-adapted viruses. Cell-culture adaptation followed by plaque purification was once a prerequisite for characterizing an RVA strain. As genome sequencing technology advances, it has become possible and a norm for RVA sequences to be determined directly from fecal specimens. It is taken for granted that mutations occur during cell-culture adaptation because high fitness in a new host (e.g., monkey kidney cells) may have been achieved at the cost of low fitness in the original host (e.g., humans). Indeed, such host range mutations often provide the molecular basis for the development of live attenuated vaccines [7]. For this reason, efforts have been made to identify the mutations that have occurred in the genome of candidate vaccine strains after many times of cell-culture passage [8,9].

Because of interests in the AU-1’s probable origin of feline rotavirus, various genes of the AU-1 strain with different passage history were sequenced over the years by different researchers on different occasions [3,10,11,12,13,14]. Such efforts, when put together, covered the entire genome of the culture-adapted AU-1 strain, but ended up with two different VP7 sequences deposited in GenBank (under accession numbers D86271 and AB792641). Each of them has been used extensively as reference in the literature [3,6]. The former reported by Wen et al. [14] belongs to the G3 lineage together with reference human strains YO and MO [15], whereas the latter reported by Gauchan et al. [5] belongs to the feline G3 lineage together with the majority of Brazilian AU-1-like strains [6]. As the AU-1 strain was distributed rather freely among researchers, the provenance and passage history of the AU-1 strain in different laboratories are largely unknown. Thus, the current study was undertaken to establish the authentic sequence of the AU-1 strain by determining the whole genome sequence of the wild-type AU-1 strain that was present in the original stool specimen (AU-1-wt) in order to determine which VP7 sequence matched that of AU-1-wt. We also aimed to determine the whole genome sequence of the triply-plaque purified seed virus (AU-1-tc) in order to identify any mutations that might have occurred during cell-culture adaptation.

## 2. Materials and Methods

Fecal specimen, 82A001, was derived from a Japanese infant with acute diarrhea seeking medical care in January 1982. She tested positive for RVA and a strain was isolated in MA104 cells which was later named AU-1 [16]. The virus was passaged five times in roller cell culture and plaque-purified three times before the seed stock (AU-1-tc) was prepared [16]. The rest of the 82A001 stool sample (hereafter, AU-1-wt) was stored frozen until 2018 when an aliquot was thawed for the Next Generation Sequencing (NGS). AU-1-wt and AU-1-tc were sequenced at the same time with an Illumina MiSeq sequencer (Illumina, San Diego, CA, USA) as described previously [17,18,19].

Briefly, the total RNA was extracted from the AU-1-wt and AU-1-tc using the TRIzol LS Reagent (Life Technologies, Grand Island, NY, USA) and a Direct-zol RNA MiniPrep Kit (ZYMO Research, USA) following the manufacturer’s protocol. A cDNA library was prepared from 100 ng of RNA using the NEBNext Ultra RNA library Prep Kit for Illumina v1.2 (New England Biolabs, Ipswich, MA, USA) and an NEBNext Multiplex Oligos for Illumina (New England Biolabs, Ipswich, MA, USA). The libraries were purified with Agencourt AMPure XP magnetic beads (Beckman Coulter, Brea, CA, USA) and assessed for quality on a MultiNA MCE-202 bioanalyzer (Shimadzu Corporation, Kyoto, Japan). NGS was performed on an Illumina MiSeq sequencer (Illumina, San Francisco, CA, USA) with a MiSeq Reagent Kit v2 (Illumina) to generate 151 paired-end reads. Using Geneious Prime^®^ software (v.2019.2.3 and v.2023.2.1), both *de novo* assembly and mapped-to-reference-based assembly were used to obtain the near-full-length of the AU-1 genome sequences. The reference sequence used for the mapped to reference assembly were: DQ490533 (VP1), DQ490536 (VP2), DQ490537 (VP3), D10970, LC178567 (VP4), DQ490538 (VP6), AB792641 (VP7), D45244 (NSP1), DQ490534 (NSP2), DQ490535 (NSP3), D89873 (NSP4), and AB008656 (NSP5) [3,5,10,11,12,13].

The contigs for each genome segment were genotyped using the Virus Pathogens Resource (ViPR) [20]. The nucleotide sequences were deposited in GenBank under accession numbers OR727616-OR727626 for AU-1-wt and OR715769-OR715779 for AU-1-tc (Table 1).

## 3. Results and Discussion

The total lengths of the AU-1-wt and AU-1-tc sequences determined in this study were 18,335 (AU-1-wt) and 18,305 (AU-1-tc) nucleotides (nt) that covered the entire coding regions of 17,397 nt in length, but that lacked a few nucleotides at the termini of the 5′-untranslated regions (Table 1).

**Figure 1:**
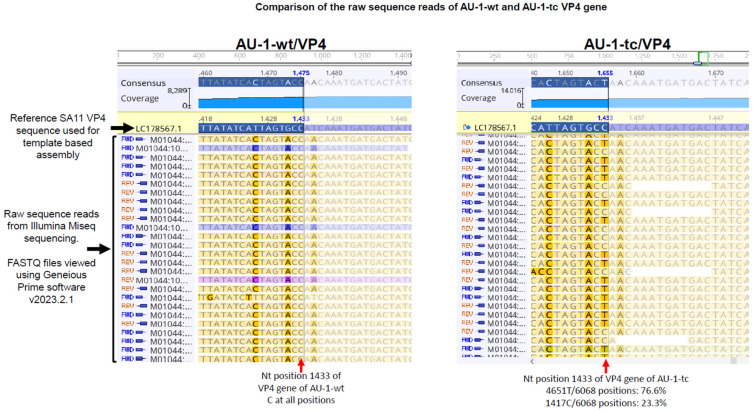
Comparison of the raw sequence reads around aa position 475 of AU-1-wt and AU-1-tc VP4 gene. Full genome sequences of AU-1-wt and AU-1-tc were generated by Illumina Miseq sequencing. FASTQ files were viewed using Geneious Prime software v2023.2.1.

The genomes of the AU-1-wt and AU-1-tc were identical except one substitution at nt position 1433 of the VP4 gene where cytosine (AU-1-wt) was replaced with uracil (AU-1-tc), resulting in a non-synonymous substitution from proline (AU-1-wt) to leucine (AU-1-tc) at amino acid (aa) position 475 (Table 2). Examination of the individual VP4 sequence reads generated from the Illumina Miseq sequencing shows that 1417 (23.4%) out of 6068 reads of AU-1-tc had cytosine at nt 1433, whereas 4651 (76.6%) had uracil (Figure 1). This indicates that there were two variants of AU-1 in the culture fluid, of which the minority were wild-type carrying P475 and the majority were mutants carrying the P475L mutation. We next examined what amino acids are present at position 475 in the VP4 of other AU-1-like strains; there was always proline at position 475 irrespective of whether the source was fecal specimens (wt) or culture-adapted strains (tc) (Table 2). Thus, the P475L mutation was considered not to be inevitable for the virus to gain higher fitness to cultured cells.

As to the conflicting entries of the AU-1 VP7 sequences in the GenBank database, the AU-1-wt sequence (OR727621) was 99.9% identical in nucleotides to the AB792641 sequence whereas it was only 92.5% identical to the D86271 sequence. Thus, the authentic AU-1 VP7 sequence belongs to the G3 feline lineage as did the VP7 sequences of the Brazilian AU-1-like strains (R47, R57, and R147) [6]. In hindsight, a closer look at the amino acid sequence deduced from the D86271 sequence appears to be different from the AU-1 VP7 aa sequence shown in the figure and tables in ref. [14]. So, it is speculated that accidental mislabeling occurred at the time of the sequence submission rather than the actual sequencing and analysis. Similarly divergent from the AU-1-wt sequence was the NSP1 gene, in which the AU-1-wt was only 91.9% identical to the one deposited in the GenBank (D45244).

The detection of only one aa substitution between the wild-type and culture-adapted AU-1 strains was unexpectedly fewer than those reported in two preceding studies [8,9]. Esona, et al. [8] found eight aa changes in five genes, including the VP4 gene at passage 27 of a G1P[8] vaccine candidate (CDC-9) isolated from feces into cell culture. Similarly, Tsugawa and Tsutsumi [9] determined the whole genome sequences of three G1P[8] strains, Wa, DC3695, and DC5685, from feces and after 60 passages in cell culture, and found 16, 16, and 14 aa changes, respectively. The scarcity of substitution in this study may be attributable to a low level of passage, which was considered fewer than 10 times including three plaque-to-plaque passages. Alternatively, strains of feline rotavirus origin may readily grow in monkey kidney cells.

## 4. Conclusions

In summary, the whole genome sequences of the AU-1-wt and AU-1-tc strains determined in this study showed that they were identical except VP4-P475L and that the VP7 gene of AU-1-wt was almost completely identical to that reported by Gauchan et al. [5]. We therefore conclude that the AU-1-wt sequences determined in this study (OR727616-OR727626) should be used as the reference. Regarding the few mutations during cell-culture adaptation, further studies are needed to see if the case of AU-1 is an exception or more generally applicable to other RVAs. To the best of our knowledge, this study was the first in which whole genome sequences of both wild-type and cultured RVA strains were simultaneously determined and analyzed by deep sequencing.

## Figures and Tables

**Table 1:** Regions determined for AU-1-wt and AU-1-tc genome segments.

Genome Segment	Reference Strain			AU-1-wt						AU-1-tc				
				Region Determined						Region Determined				
	Length			Start	End	Length (Base Pairs)	% of Length	Accession #		Start	End	Length (Base Pairs)	% of Length	Accession #
VP1	3302	AU-1		11	3302	3292	99.70	OR727616		9	3302	3294	99.76	OR715769
VP2	2708	AU-1		11	2708	2698	99.63	OR727617		11	2703	2693	99.45	OR715770
VP3	2591	AU-1		10	2591	2582	99.65	OR727618		10	2583	2574	99.34	OR715771
VP4	2359	AU-1		10	2359	2350	99.62	OR727619		10	2359	2350	99.62	OR715772
VP6	1357	AU-1		1	1357	1357	100.00	OR727620		15	1355	1341	98.82	OR715773
VP7	1063	SA11		12	1052	1052	98.97	OR727621		13	1056	1044	98.21	OR715774
NSP1	1611	SA11		12	1598	1555	96.52	OR727622		12	1598	1555	96.52	OR715775
NSP2	1059	SA11		11	1059	1049	99.06	OR727623		33	1052	1020	96.32	OR715776
NSP3	1073	AU-1		1	1070	1070	99.72	OR727624		10	1066	1057	98.51	OR715777
NSP4	751	SA11		1	673	673	89.61	OR727625		13	738	725	96.54	OR715778
NSP5	667	AU-1		11	667	657	98.50	OR727626		11	662	652	97.75	OR715769
Total	18,541					18,335						18,305		

**Table 2:** Comparison of amino acid positions of AU-1 P[9] VP4 to other P[9] and P-genotypes.

Category	Strain	P-Type	Amino Acid Position in VP4											Reference/Accession Number
			470	471	472	473	474	475	476	477	478	479	480	
Human-wt P[9]	RVA/Human-tc/JPN/AU-1/1982/G3P[9]	P[9]	L	I	S	L	V	L	T	N	D	D	Y	This study
	RVA/Human-tc/JPN/AU-1/1982/G3P[9]	P[9]	.	.	.	.	.	.	.	.	.	.	.	D10970.1
	RVA/Human-wt/JPN/AU-1/1982/G3P[9]	P[9]	.	.	.	.	.	P	.	.	.	.	.	This study
	RVA/Human-wt/CHN/L621/2006/G3P[9]	P[9]	.	.	.	.	.	P	.	.	.	.	.	EU708574
	RVA/Human-wt/THA/CU365-KK/2008/G3P[9]	P[9]	.	.	.	.	.	P	.	.	.	.	.	JN706511.1

Human-tc P[9]	RVA/Human-tc/JPNAU1115/1986/G3P[9]	P[9]	.	.	.	.	.	P	.	.	.	.	.	LC328184.1
	RVA/Human-tc/JPN/AU938/1989/G3P[9]	P[9]	.	.	.	.	.	P	.	.	.	.	.	LC328185.1
	RVA/Human-tc/BRA/R57/1997/G3P[9]	P[9]	.	.	.	.	.	P	.	.	.	.	.	KJ820861.1
	RVA/Human-tc/BRA/R142/1999/G3P[9]	P[9]	.	.	.	.	.	P	.	.	.	.	.	KJ820906.1
	RVA/Human-tc/KOR/CAU14-1-262/2014/G3P[9]	P[9]	.	.	.	.	I	P	.	.	.	.	.	KR262152.1

Cat-wt P[9]	RVA/Cat-wt/THA/Meesuk/2021/G3P[9]	P[9]	.	.	.	.	.	P	.	.	.	.	.	ON191599.1
	RVA/Cat-wt/ITA/BA222/2005/G3P[9]	P[9]	.	.	.	.	.	P	.	.	.	.	.	GU827409.1

Cat-tc P[9]	RVA/Cat-tc/AUS/Cat2/1984/G3P[9]	P[9]	.	.	.	.	.	P	.	.	.	.	.	EU708959
	RVA/Cat-tc/JPN/FRV-1/1986/G3P[9]	P[9]	.	.	.	.	.	P	.	.	.	.	.	D10971.1
	RVA/Cat-tc/JPN/FRV317/1994/G3P[9]	P[9]	.	.	.	.	I	P	.	.	.	.	.	LC328214.1
	RVA/Cat-tc/JPN/FRV381/1994/G3P[9]	P[9]	.	.	.	.	I	P	.	.	.	.	.	LC328214.1
	RVA/Cat-tc/JPN/FRV384/1994/G3P[9]	P[9]	.	.	.	.	I	P	.	.	.	.	.	LC328215.1

Other P-types	RVA/Human-lab/USA/Wa/1974/G1P[8]/virulent	P[8]	.	.	.	.	.	P	.	.	.	.	.	MT025868.1
	RVA/Human-lab/USA/Wa/1974//G1P[8]/attenuated	P[8]	.	.	H	.	.	P	.	.	.	.	.	MT025869.1
	RVA/Vaccine/USA/Rotarix/1988//G1P[8]	P[8]	.	.	.	.	.	P	.	.	.	.	.	JN849113.1
	RVA/Pig-tc/USA/OSU/1977/G5P9[7]	P[7]	.	.	.	.	.	P	S	.	.	.	.	MT025934.1
	RVA/Human-tc/AUS/RV3/1977/G3P[6]	P[6]	.	.	.	.	.	P	S	.	.	.	.	U16299.1
	RVA/Human-wt/USA/DS-1/1976/G2P[4]	P[4]	.	.	.	.	.	P	.	.	.	.	.	MT796872.1
	RVA/Human-wt/USA/DS-1/1976/G2P[4]	P[4]	.	.	.	.	.	Q	.	.	.	.	.	AJ540227.1
	RVA/Human-tc/USA/DS-1/1976/G2P[4]	P[4]	.	.	.	.	.	Q	.	.	.	.	.	HQ650119.1
	RVA/Human-tc/USA/DS-1/1976/G2P[4]	P[4]	.	.	.	.	.	Q	.	.	.	.	.	EF672577.1
	RVA/Simian-tc/USA/RRV/1975/G3P[3]	P[3]	.	.	.	.	.	P	S	.	.	.	.	HQ846846
	RVA/Simian-tc/ZAF/SA11/1958/G3P[2]	P[2]	.	.	.	.	.	P	S	.	.	.	.	D16345.1
	RVA/Cow-tc/USA/NCDV/1971/G6P[1]	P[1]	.	.	.	.	.	P	S	.	.	.	.	AFC40967.1

## Data Availability

The data presented in this study are openly available in GenBank/DDBJ/EMBL under the accession numbers: OR727616-OR727626; OR715769-OR715779. [GenBank/DDBJ/EMBL] [https://www.ncbi.nlm.nih.gov/nuccore/AU-1-wt_AU-1-tc] [OR727616-OR727626; OR715769-OR715779].
